# Light-Chain (AL) Cardiac Amyloidosis Presenting as Heart Failure With Reduced Ejection Fraction

**DOI:** 10.7759/cureus.55271

**Published:** 2024-02-29

**Authors:** Luis E Santiago, Ali Tariq Alvi, Veniamin Melnychuk, Philip Mesquita, Pallavi Aneja

**Affiliations:** 1 Internal Medicine, HCA Florida Northwest Hospital, Margate, USA; 2 Internal Medicine, HCA Florida Westside Hospital, Plantation, USA

**Keywords:** ttr cardiac amyloidosis, cardiac amyloidosis with reduced ejection fraction, diastolic heart failure, cardiac amyloidosis, immunoglobulin light-chain amyloidosis

## Abstract

Systemic amyloidosis is caused by the extracellular deposition of misfolded proteins in various organs and usually leads to organ dysfunction. The two common subtypes include light-chain amyloidosis and transthyretin amyloidosis. Deposition of these proteins in the heart can lead to infiltrative and restrictive cardiomyopathy, commonly manifesting as heart failure with preserved ejection fraction. However, systolic heart failure with reduced ejection fraction is mainly seen in the advanced stages of the disease. Here, we present the case of a 53-year-old female who presented with new-onset heart failure with reduced ejection fraction with no prior symptoms or diagnosis of amyloidosis and diastolic dysfunction.

## Introduction

Cardiac amyloidosis is a rare condition caused by the accumulation of amyloid fibrils within the extracellular space of the heart. Approximately 95% of cardiac amyloidosis cases are caused by the deposition of transthyretin (TTR) or immunoglobulin light chains [1-3]. It affects about 1 in every 100,000 people annually in the United States [4]. Clinical symptoms vary greatly depending on which organs are involved, often presenting as non-specific complaints, which can lead to delays in diagnosis. Both diastolic as well as systolic impairment are present, although the left ventricular ejection fraction (LVEF) is typically normal until advanced stages, in which it is typically only mildly reduced [5]. Left untreated, patients with cardiac light-chain amyloidosis (AL) and heart failure have a grim prognosis, with overall median survival as brief as six months [6]. However, due to advances in management, the outlook for those with AL amyloidosis and cardiac involvement has significantly improved, with a median survival of approximately 5.5 years post-diagnosis [7]. We present the case of a 53-year-old female who presented with new-onset heart failure with a reduced ejection fraction of 35% with no prior symptoms or diagnosis of amyloidosis or diastolic dysfunction.

## Case presentation

**Figure 1:**
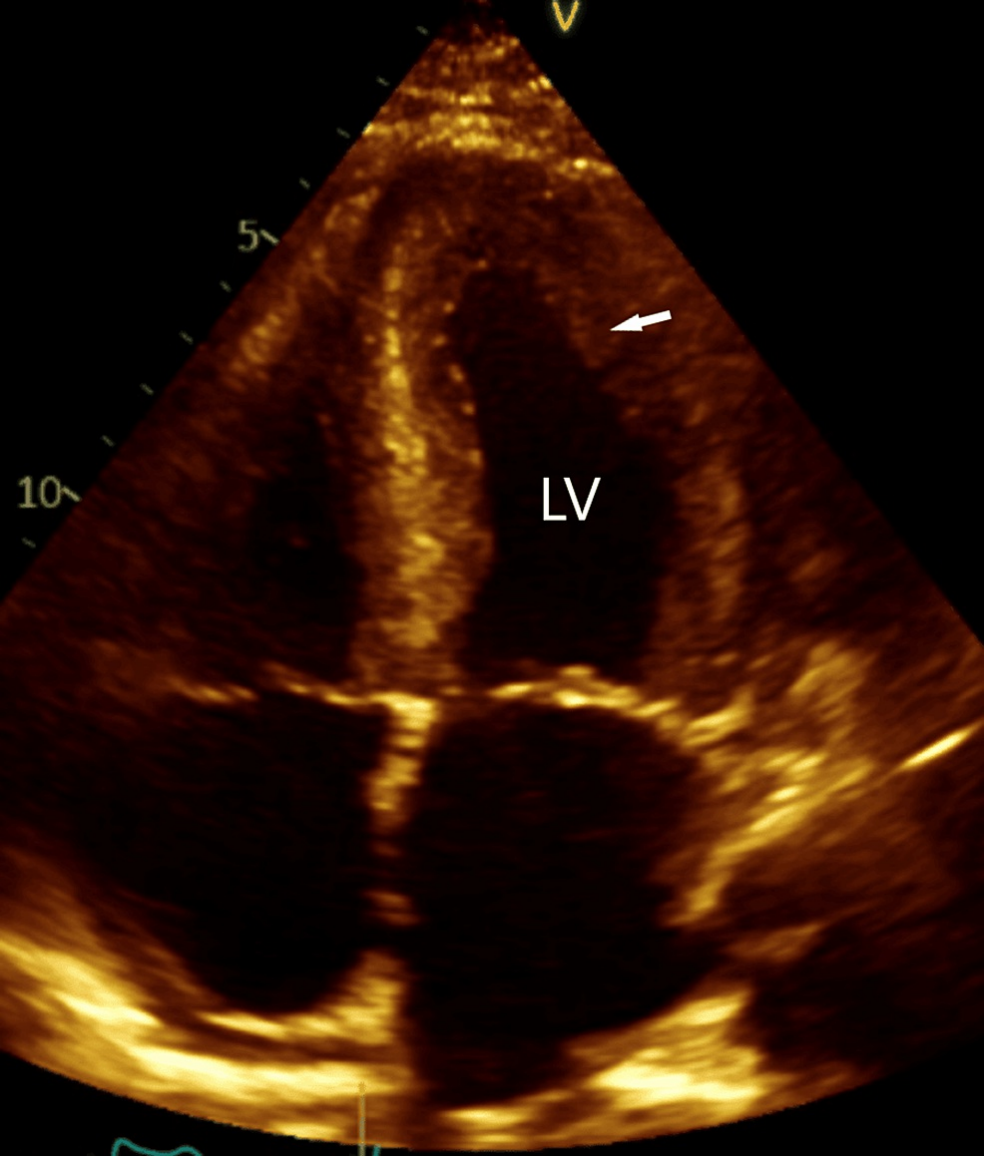
Four-chamber echocardiogram view. Four-chamber echocardiogram view showing Increased left ventricular wall thickness (arrow). LV: left ventricle

**Figure 2:**
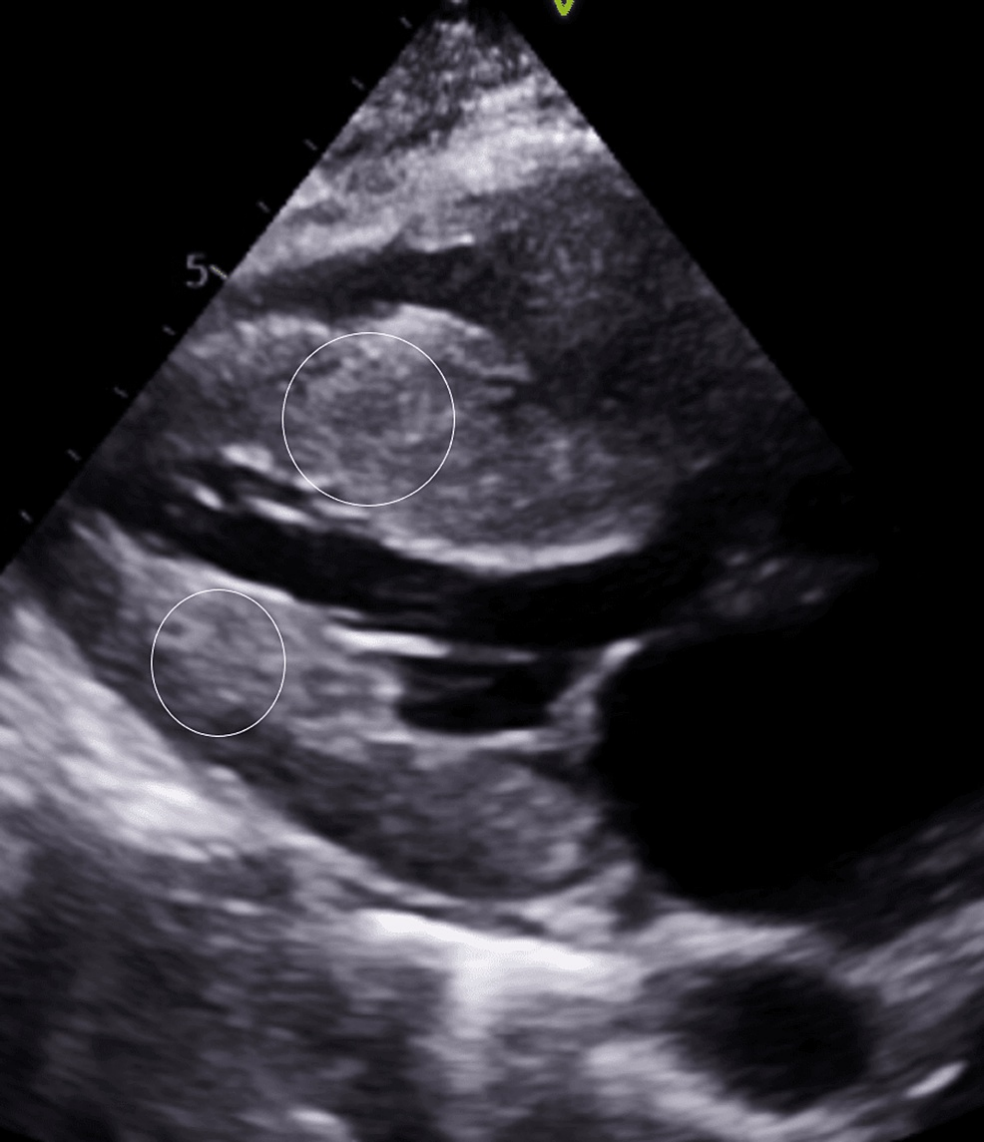
Parasternal long-axis echocardiogram view. Parasternal long-axis echocardiogram view showing increased left ventricular wall thickness (circles).

A 53-year-old African American female with a past medical history of essential hypertension and asthma presented to the emergency department with worsening shortness of breath, decreased appetite, and fatigue for three weeks. She denied recent weight loss, chills, night sweats, fever, cough, or chest pain. In the emergency department, she was afebrile, with a heart rate of 101 beats per minute, blood pressure of 115/83 mmHg, oxygen saturation of 97% on room air, and respiratory rate of 25 breaths per minute. Laboratory investigations are presented in Table 1. Due to elevated D-dimer, we performed computed tomography angiography of the chest, which revealed bilateral pulmonary emboli with no evidence of right ventricular strain and subtle lucent lesions in the bones concerning for malignancy. Therefore, we started anticoagulation with enoxaparin and ordered a laboratory workup for multiple myeloma. Serum protein electrophoresis was unremarkable. The free kappa/lambda ratio was 1,411.94, which was diagnostic of light-chain myeloma. Transthoracic echocardiogram showed an LVEF of 35% and moderately increased wall thickness (Figures 1, 2).

**Table 1:** Laboratory results.

Lab test	Result	Reference range
White blood cell count	81.0 × 10^9^/L	4.0–11.0 × 10^9^/L
Hemoglobin	10.9 g/dL	12.0–16.0 g/dL
Platelet count	351.0 × 10^9^/L	150–450 × 10^9^/L
Sodium	140 mmol/L	135–145 mmol/L
Potassium	4.5 mmol/L	3.5–5.0 mmol/L
Chloride	106 mmol/L	98–107 mmol/L
Bicarbonate	24 mmol/L	22–29 mmol/L
Blood urea nitrogen	13 mg/dL	7–20 mg/dL
Creatinine	0.74 mg/dL	0.6–1.1 mg/dL
Albumin	3.9 g/dL	3.5–5.0 g/dL
NT-proBNP	10,900 pg/mL	<300 pg/mL (may vary by age/gender)
Calcium	10.7 mg/dL	8.5–10.5 mg/dL
Prothrombin time	15.1 seconds	11.0–13.0 seconds
International normalized ratio	1.3	0.9–1.1
Activated partial thromboplastin time	28 seconds	25–35 seconds
D-dimer	6,373 ng/mL	<500 ng/mL (may vary by laboratory)
IgG	361 mg/dL	700–1,600 mg/dL
IgA	<40 mg/dL	70–400 mg/dL
IgM	<25 mg/dL	40–230 mg/dL
Free kappa light chains	5,083 mg/L	3.3–19.4 mg/L
Free lambda light chains	3.6 mg/L	5.7–26.3 mg/L
Free kappa/lambda ratio	1,411.94	0.26–1.65 (ratio)

**Figure 3:**
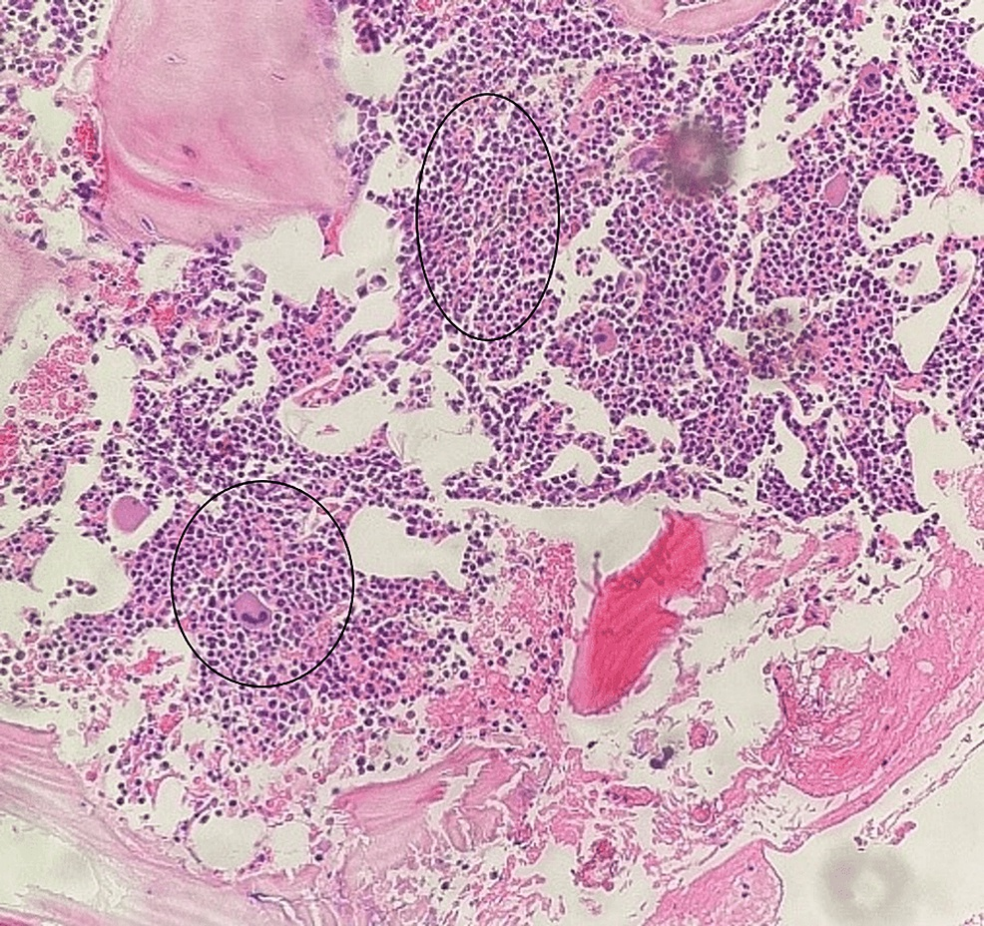
Bone marrow biopsy. Hematoxylin and eosin staining showing diffuse infiltration of bone marrow by plasma cells (circles).

**Figure 4:**
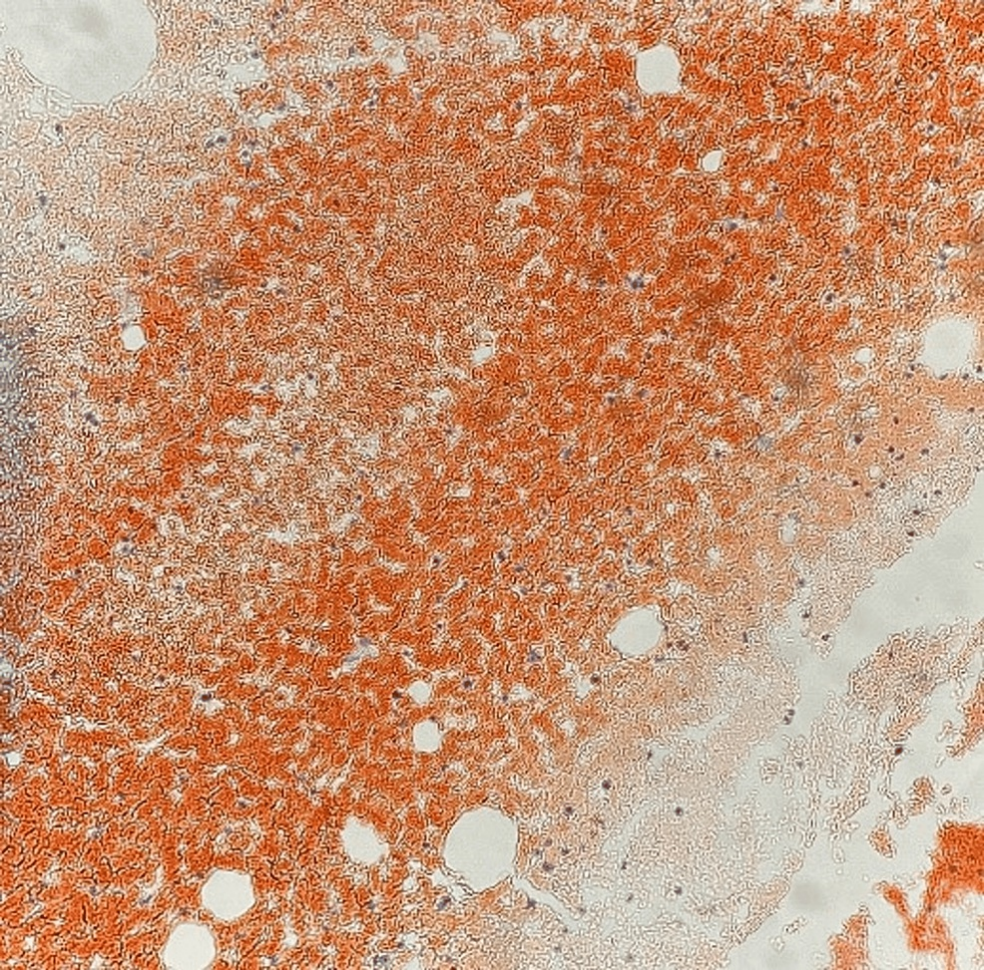
Bone marrow biopsy. Congo red stain showing amyloid deposits in bone marrow.

**Figure 5:**
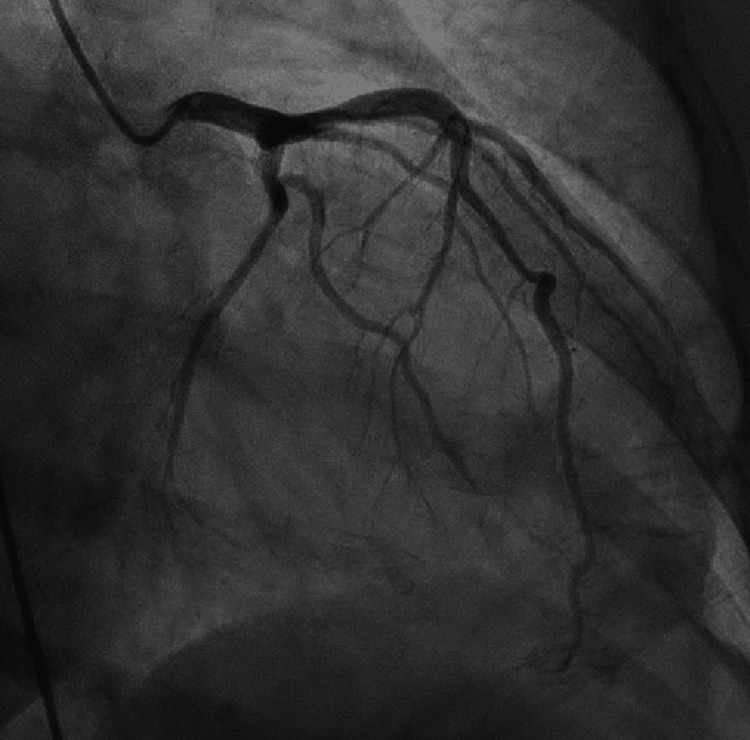
Left anterior oblique caudal image from left heart catheterization. Left anterior oblique caudal view from left heart catheterization showing angiographically normal left coronary arteries.

**Figure 6:**
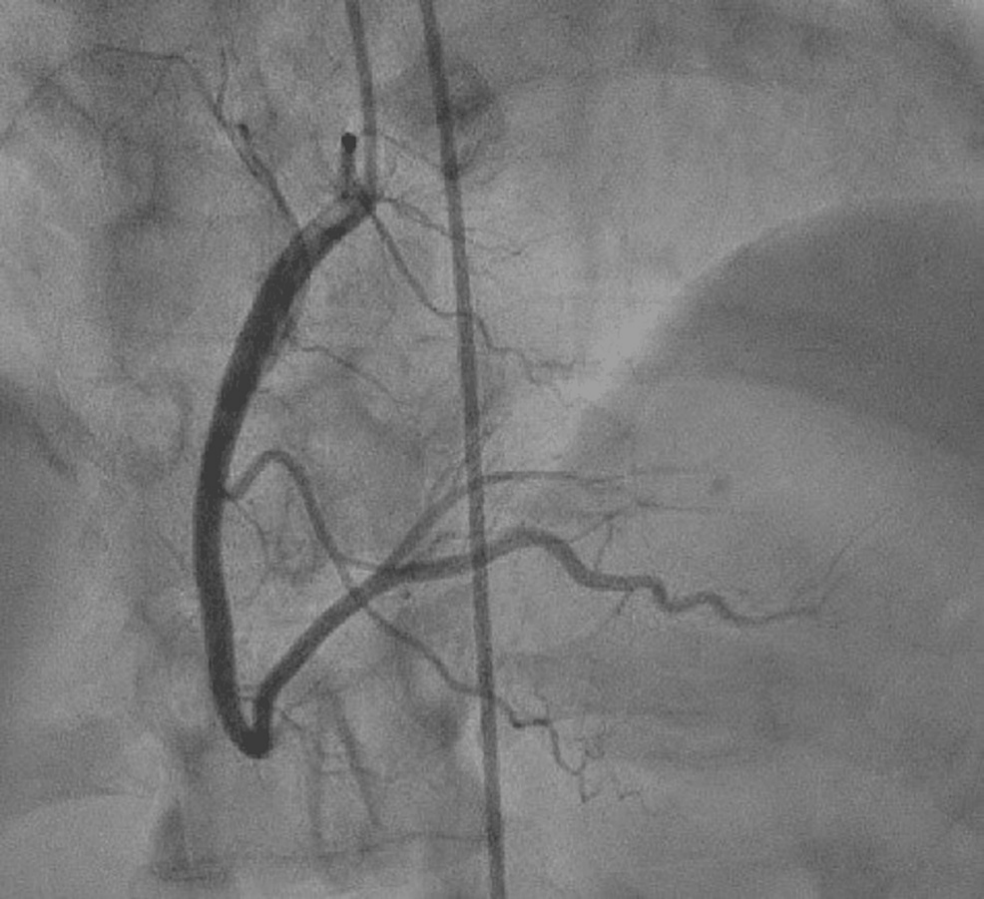
Left anterior oblique caudal image from left heart catheterization. Left anterior oblique caudal image from left heart catheterization showing angiographically normal right coronary arteries.

We ordered a bone marrow biopsy, which revealed hypercellular bone marrow with extensive infiltration by plasma cells, comprising about 80% of the bone marrow (Figure 3). It also showed amyloid fibrils on hematoxylin and eosin-stained and congo red-stained biopsy sections (Figure 4). Pyrophosphate myocardial scan (99mTc-PYP) showed no abnormal uptake to suggest cardiac transthyretin amyloidosis (ATTR). After the confirmation of diagnosis, she was started on a chemotherapy regimen with cyclophosphamide, bortezomib, and dexamethasone. We also started guideline-directed medical therapy for heart failure with reduced ejection fraction (HFrEF). Left heart cardiac catheterization with coronary angiography was performed to rule out coronary artery disease. This showed angiographically normal coronaries (Figures 5, 6).

## Discussion

Cardiac amyloidosis is an underdiagnosed cause of cardiomyopathy causing heart failure with preserved ejection fraction. ATTR amyloidosis is caused by misfolded TTR deposition, a protein tetramer produced by the liver and deposited in the extracellular space as insoluble amyloid, resulting in damage to the peripheral nerves and heart. ATTR cardiac amyloidosis can be wild type (ATTRwt-CA) caused by age-related misfolding of unaltered TTR or a variant type (ATTRv-CA) caused by an autosomal dominant mutation in the gene involving the TTR protein. ATTRv-CA can present as familial amyloid polyneuropathy, familial amyloid cardiomyopathy, or an overlap of both. It is usually diagnosed at a young age with neuropathy (i.e., carpal tunnel syndrome), typically preceding cardiomyopathy. Cardiac involvement in both types commonly presents as heart failure with preserved ejection fraction; however, HFrEF can also develop in later stages of the disease [8,9].

AL amyloidosis is a rare condition and can affect almost every organ in the body, resulting in multiorgan failure. The clinical course of this type is more virulent and accelerated than ATTR amyloidosis. The red flag features for cardiac amyloidosis include low QRS voltage on electrocardiogram along with specific echocardiogram findings, including diastolic dysfunction with a restrictive pattern, atrial enlargement, speckled myocardium, and increased left ventricular wall thickness [8,9]. Moreover, apical sparing of longitudinal strain is considered sensitive and specific in differentiating cardiac amyloidosis from other causes of left ventricular hypertrophy [10]. In this patient, the echocardiogram showed left ventricular hypertrophy but no apical sparing of longitudinal strain and a speckled myocardium. Cardiac magnetic resonance imaging is another useful imaging technique that can show cardiac amyloidosis by late gadolinium enhancement, but it also does not differentiate between AL and ATTR subtypes [11]. Nuclear imaging techniques with bone-seeking radiotracers have become the diagnostic test of choice to differentiate the two subtypes, with 99mTc-PYP being commonly used in the United States [9,12]. In our case, 99mTc-PYP showed no abnormal uptake, ruling out ATTR [8,9]. Next, with AL amyloidosis, we need to evaluate for a plasma cell disorder by testing for serum and urine protein electrophoresis and serum-free light chains. The presence of a plasma cell dyscrasia on these tests points toward AL amyloidosis, which then requires confirmation with biopsy [13].

Management in cardiac amyloidosis is aimed at maintaining euvolemia by sodium restriction and diuretic use. Treatment of ATTR cardiac amyloidosis involves using ATTR silencers that prevent transthyretin synthesis and ATTR stabilizers that prevent transthyretin tetramer dissociation into monomers. Management of AL cardiac amyloidosis is similar to that of multiple myeloma, involving the use of anti-plasma cell therapy and autologous stem cell transplant. Anti-plasma therapy includes immunomodulatory drugs, proteasome inhibitors, and monoclonal antibodies against plasma cell surface antigens [8]. In our patient, we used the same strategy by using cyclophosphamide and bortezomib, along with dexamethasone.

## Conclusions

Cardiac amyloidosis remains a rare disease despite the recent increase in diagnosis owing to improved imaging techniques, increased physician suspicion, and the fact that endomyocardial biopsy is no longer required for diagnosis. As the frequency of cardiac amyloidosis continues to increase, we believe the prevalence of cases with systolic heart failure will also rise. The patient that we have described in this case report developed systolic heart failure concomitantly with a diagnosis of light-chain myeloma. This case report highlights the possibility of having HFrEF in patients with AL cardiac amyloidosis, and it should alert clinicians to consider it as a diagnostic differential.
